# Preference for Curvature: A Historical and Conceptual Framework

**DOI:** 10.3389/fnhum.2015.00712

**Published:** 2016-01-12

**Authors:** Gerardo Gómez-Puerto, Enric Munar, Marcos Nadal

**Affiliations:** ^1^Human Evolution and Cognition Group, IFISC, University of the Balearic Islands—CSICPalma, Spain; ^2^Department of Basic Psychological Research and Research Methods, University of ViennaVienna, Austria

**Keywords:** preference, curvature, angularity, empirical aesthetics, evolutionary aesthetics

## Abstract

That people find curved contours and lines more pleasurable than straight ones is a recurrent observation in the aesthetic literature. Although such observation has been tested sporadically throughout the history of scientific psychology, only during the last decade has it been the object of systematic research. Recent studies lend support to the idea that human preference for curved contours is biologically determined. However, it has also been argued that this preference is a cultural phenomenon. In this article, we review the available evidence, together with different attempts to explain the nature of preference for curvature: sensoriomotor-based and valuation-based approaches. We also argue that the lack of a unifying framework and clearly defined concepts might be undermining our efforts towards a better understanding of the nature of preference for curvature. Finally, we point to a series of unresolved matters as the starting point to further develop a consistent research program.

## Introduction

Curved lines and forms occupy a special place in the Western traditions of philosophical, psychological, and evolutionary thought on aesthetics (e.g., Hogarth, 1753; Spencer, 1873; Allen, 1877; Santayana, 1896; Valentine, 1913). They have often been regarded as more harmonious, relaxing, or pleasant—and more in consonance with nature—than straight or broken lines. Only after the development of Fechner’s (1876) empirical aesthetics, however, were such conjectures about curvature subjected to experimental scrutiny. Stratton’s (1902) attempt to relate the pleasure derived from the observation of curved lines to the concurrent movements of the extraocular muscles constitutes one of the earliest empirical tests of the contribution of curvature to aesthetic experience. Like Spencer (1873) and Santayana (1896) before him, Stratton (1902) conjectured that eye movements required to follow sharp, broken lines must be more abrupt and, therefore, less pleasant than those required to follow curved lines. Using an early eye-tracking device, Stratton (1902) recorded the gaze patterns of two participants while viewing different kinds of curved stimuli and test this hypothesis.

The jerky and discontinuous nature of saccades, even when participants attempted to follow smooth curved lines, proved his original expectation wrong. Nevertheless, Stratton’s (1902) study greatly influenced subsequent research on the aesthetic qualities of curvature (Valentine, 1913). In fact, his seminal work included reflections on the two issues that have been the central focus of research and discussion during the 20th and 21st centuries: (i) the mechanism underlying preference for curvature and (ii) the functional significance of such preference. On the one hand, he believed that curved lines provide observers with a continuous flow of information that is easy to process. On the other hand, he noticed that experience, environment, and cultural cues might influence the appraisal of those lines. He pointed out that most movements in nature are curved, which makes us perceive the curved line as an indication of a functional, normal behavior. But it is not only in curved lines that we find meaning: while curved lines might be more appealing due to their complete and perfect nature, broken lines, though imperfect, convey a stronger sense of power.

Although research on preference for curvature was conducted only sporadically during most of the 20th century, interest in the cognitive and neural mechanisms underpinning preference for curvature, as well as in its psychological and biological functions, resurged in the last decade (e.g., Bar and Neta, 2006, 2007; Leder et al., 2011; Vartanian et al., 2013; Bertamini et al., 2015). Nevertheless, the mere accumulation of behavioral and neuroimaging data does not ensure the progress of scientific research. Explaining the cognitive and biological mechanisms underlying preference for curvature requires, just like in other domains of the cognitive sciences (Block, 2014)—and science in general–, substantive theories resting on clear concepts.

In this article, we develop a framework for research on the psychological and neural mechanisms involved in preference for curvature. We follow the history of this research and the conceptual unfolding of the two main issues raised in Stratton’s (1902) discussion. First, regarding the mechanisms underlying humans’ preference for curvature, we distinguish approaches that base their explanation on features of the sensorimotor systems from those that base it on appraisal processes. Second, regarding the origin of this preference, we distinguish approaches positing an evolutionary foundation from those postulating it is the result of learning processes.

## Preference for Curvature: Cognitive and Neural Mechanisms

### Sensorimotor-Based Explanations

A number of researchers have argued that human preference for curvature derives from the way in which physical properties of curved stimuli directly interact with specific characteristics of the sensorimotor system. Thus, from this perspective, preference for curved features owes to a sort of natural coupling between perceptual features and sensorimotor processes that are attuned to curved configurations. This general framework has been developed in three directions, differing in their central explanatory mechanism: movement, specific neural activity, and fluency/Gestalt principles.

#### Movement

Stratton’s aforementioned studies attempted to explain the perceived beauty of curved stimuli as a consequence of sensorimotor activity, namely eye movements. This approach was inspired by Spencer’s (1873) idea that the grace of curved lines is enjoyable because it gives a sense of economy in the expenditure of force, and by Wundt’s notion that the pleasure provided by curves resulted from the ease of the eyes’ motion as they glided over the curve. However, as noted above, Stratton’s results did not support these conjectures. Whereas the line offered to the eyes for following was continuous and smooth, the gaze path itself was irregular, varying, and even sharp angled. Even when the eyes follow a curve, their movements are characterized by jerks and pauses, short rapid flights followed by sudden interruptions. Stratton finally concluded that it would be an error to regard the enjoyment of graceful forms as resulting from this muscular adjustment, and considered other alternatives beyond the simple sensuous impression, whether muscular or retinal.

A different possibility is that preference for curvature is not related to the movements of the eyes, but to the ease and comfort of certain movements when drawing, or simply moving our arms or hands. As stated by Hogarth (1753, p. 38) in his classical *Analysis of Beauty*: “It is to be observed […] that the waving line, or line of beauty, varying still more, being composed of two curves contrasted, becomes still more ornamental and pleasing, insomuch that the hand takes a lively movement in making it with pen or pencil.” There seems to be, however, little or no empirical research addressing the relation between preference for lines and the ease of movements required to produce them. For instance, although Martin (1906) considered Hogarth’s observation as an alternative hypothesis to Stratton’s initial conjecture, she did not fully explore those thoughts empirically.

#### Neural Activity

Preference for curvature has also been explained in terms of the neurophysiology of the visual system. Fantz and Miranda (1975), for instance, showed that 1-week-old neonates fixate longer on curved contour geometric forms than on sharp contour ones. They explained this very early preference for curvature based on Hubel and Wiesel’s (1968) identification of a set of cortical cells whose activity is sensitive to deviations from continuous straight contours, such as curves and angles. Fantz and Miranda (1975) suggested that the differential fixation on curved and straight geometric forms might be related to these cells’ responsiveness to shifts in line direction, with curved visual patterns inducing greater activity in these cells than straight patterns. Although it is not clear how this differential activity might translate into differences in looking time, the neural coding hypothesis has recently gained traction after it has been shown that preference for curvature is still found when participants are presented with stimuli consisting of the low spatial frequencies of images depicting real objects—but not when those stimuli contain only the high spatial frequencies of the original images (Bar and Neta, 2007). This is consistent with Vuilleumier et al.’s (2003) finding that high and low spatial frequency information in visual images is processed by distinct neural channels. They showed dissociable roles of these channels for processing emotional expressions. Low-frequency faces with fearful expressions elicit greater responses in specified subcortical pathways (amygdala, pulvinar and superior colliculus) than the same high-frequency faces.

#### Fluency and the Gestalt Principles

Quinn et al. (1997) showed that Gestalt organizational effects and preference for curvature are both involved in the initial parsing and subsequent organization of complex visual patterns. Using a familiarization-novelty preference procedure, the authors found that 3- and 4-month-old infants were able to segregate the contours of two intersecting visual forms, and that they did so relying on the Gestalt principle of good continuation. Moreover, they argued that spontaneous preference for curvature facilitated the Gestalt organization of complex configurations into coherent forms.

This explanation is related to the processing fluency theory of aesthetic pleasure (Reber et al., 2004): fluent processing of an object leads to positive aesthetic responses. From this perspective, preference for curved stimuli is greater than preference for non-curved stimuli because curvature facilitates processing fluency. Indeed, there are several studies that have reported that curvilinear features are easier to detect (Treisman and Gelade, 1980; Wolfe et al., 1992; Álvarez et al., 2002). However, Bar and Neta (2006, 2007) found no differences in the time it took participants to rate curved and sharp stimuli, even when curved ones were preferred. This led them to conclude that curved features did not facilitate the processing of the stimuli and therefore, the explanation for preference should lie elsewhere.

Nonetheless, the time participants take to respond in a preference task need not correspond to the speed of processing curves or sharp angles. Ruta et al. (2014) found that participants are faster in detecting intrinsic features of curved polygons compared to their angular version, which seems to indicate that efficient visual processing is affected by the presence of curved features in the contour. They also explored the relation between the global/local configuration of the stimulus and preference for curvature, finding that preference remained even with the local elements being orthogonal to the continuity of the global contour. This led them to conclude that preference for curvature is likely caused by intrinsic characteristics of the lines, which can be described as cases of good continuation or good Gestalt.

### Appraisal-Based Explanations

A good amount of research on preference for curvature has focused on appraisal processes, whether implicit or explicit, and the way in which they impact aesthetic experience. In contrast to the different sensorimotor-based approaches noted above, appraisal approaches show strong consistency and thematic uniformity, motivated by early findings about the emotional evaluation of straight and curved lines. Even when these studies report slight differences in the qualities and connotations ascribed to angular features, they tend to agree that curvature is imbued with non-representational semantic meaning.

In Lundholm’s (1921) early study, eight participants received a series of words describing different kinds of feelings and were asked to draw lines matching those words. Results showed that sharp lines were considered to be *agitating, hard*, or *furious*, whereas curved ones were perceived as *gentle* and *quiet*—but also *sad* or *lazy*. Extending this research line, Poffenberger and Barrows (1924) asked 500 adult participants to perform the inverse task: to match those lines drawn by Lundholm’s participants to a given list of feelings. Their results confirmed Lundholm’s (1921) earlier findings, as did Hevner’s (1935), who used a more complex set of stimuli that included not only lines, but also abstract shapes.

Although this exploration of the links between feelings and different degrees of angularity was both relevant and promising, empirical interest in curvature seemed to fade somewhat in the following decades. Later work, including Guthrie and Wiener’s (1966) and Kastl and Child’s (1968) research, moved beyond the study of the effects of isolated lines, and analyzed the impact of the curvature of objects’ contours.

Guthrie and Wiener (1966) sought to prove that the response to subliminal stimuli was not determined by observers’ full discrimination and comprehension of the presented stimuli. They believed, rather, that observers relied mainly on primitive cues that elicited a predicable response. Their initial results showed that participants’ responses varied when presented with lines differing in their angularity and thickness, with curved lines being linked to positive traits, and sharp ones to negative traits. In order to further explore the possibility that the feelings ascribed to isolated lines were responsible for differing reactions to more complex subliminal stimuli, they prepared two very similar drawings of a sitting man. In one of them, the man was pointing a gun to his head; in the other he was not. After making sure that participants were able to discriminate the valence of the image when presented subliminally, Guthrie and Wiener (1966) created two different versions of each image: one in which the overall contour was sharp, and another in which it was smooth. This allowed them to prove that, as predicted, it was the overall sharpness, and not the presence of a gun, that determined whether the image was perceived as threatening and negative.

Kastl and Child’s (1968) study of the emotional meaning of different typographies lent additional support to the notion that the distinct feelings conveyed by isolated lines remains relevant when the stimuli presented consists of more complex shapes and contours. Still, it should be noted that some of their findings, such as angular types appearing to be *sadder*, contradict those of Lundholm’s (1921) work.

Research on the effects of curvature on preference was reinvigorated by Bar and Neta’s (2006) study, designed to test the hypothesis that curved stimuli are preferred because sharp contours evoke a sense of threat. They first presented a sample of 14 participants with images depicting abstract shapes and everyday objects varying in the curvature of their contour. Each image was shown for only 84 ms, and participants were asked to make a like/dislike choice. Their results revealed that curved stimuli were liked more than neutral or sharp ones (Bar and Neta, 2006). In a subsequent neuroimaging study, they again found higher liking for stimuli with curved contours, and that sharp contours were subjectively perceived as more threatening. Moreover, they observed a bilateral increase in amygdala activity when participants were presented with sharp stimuli, as was expected if such stimuli did elicit a sense of threat (Bar and Neta, 2007).

Leder et al. (2011) further explored this interaction between preference for curvature and threat perception. They hypothesized that if the perception of threat produced by an object was related to preference, then the negative valence of an object could override other positive cues such as the curvature of its contour. In order to test this assumption, and after replicating the findings of preference using the same stimuli as Bar and Neta (2006), they presented participants with new stimuli depicting images of real objects that had been manipulated to create a round and a sharp contoured version of each of the 20 selected objects. These objects were selected according to their emotional valence, so that they could be evenly split into two groups depending on whether their valence was positive or negative. As predicted, they again found that curved stimuli were preferred to sharp ones, but only when the objects had a positive or neutral valence. They argued that this shows how threat and preference are interconnected, and that semantic evaluation takes precedence over the evaluation of contour, overriding the effects that curvature might have in preference.

In contrast to these studies, there is growing evidence questioning the notion that curvature is preferred because sharp angles are perceived as threatening. Bertamini et al. (2015), asked 36 participants to perform a manikin task in which they had to bring a stick figure closer or farther, as instructed, from a series of irregular polygons varying in their curvature. The study showed that participants moved the manikin faster when presented with sharp stimuli, independently of whether they were instructed to bring it closer or farther. But when presented with curved stimuli, participants reacted faster only when the task was to bring it closer to the polygons. This is the opposite behavior that would be expected if sharp angles were perceived as threatening. Thus, Bertamini et al. (2015) concluded that preference owes to the intrinsic characteristics of curvature, be it configurational or featural, and not to a rejection of sharp contours.

In the same line, Vartanian et al.’s (2013) fMRI study, in which participants were presented with images of interior architectural spaces, found no increase in amygdala activity when viewing photographs of rooms with sharp angled contours, and that curved spaces were subjectively preferred overall. The authors suggested that this finding could be explained by sharp cues in buildings having lost their threatening nature by learning and exposure. This raises the fundamental point of how much preference for curvature, if any, might be culturally determined.

## Preference for Curvature: Origins

### A Learnt Preference

By being more familiarized with a certain kind of feature, or by having specific expectations regarding the shape of a given object, we can process it easier and faster, which in turn will cause that feature or shape to be preferred to others (Bornstein and D’Agostino, 1994). Familiarity and mere exposure are indeed the factors that Leder and Carbon (2005) used to explain the results from their research on car design. In their study, participants were presented with drawings of car interiors varying in a series of dimensions, such as innovation, complexity, or form. Whereas curved interiors were preferred to straight ones, the authors argued that, in this particular field of design, straight lines were innovative; and therefore, the mere exposure effect could be held accountable for effects of preference for curvature. Carbon (2010) sought to further explore this possibility by showing participants images of automobiles representing different epochs and styles in car design. His results showed that curved car exteriors were only preferred when the design itself belonged to a decade in which the trend was to build a more rounded chassis. This, he claimed, showed that preference for curvature was not static and uniform, but it was under the influence of the aesthetic *Zeitgeist* of a given time.

The unclear—but significant—link between expertise and liking of curved shapes reported by Silvia and Barona (2009) may be considered further proof of preference for curvature being mediated by cultural learning and experience. When participants with different artistic expertise were shown a series of stimuli that consisted of a differing number of hexagons and circles, only non-expert ones found circles to be more pleasant, with experts preferring even slightly more the stimuli depicting hexagons. In a second experiment, in which abstract shapes, varying in curvature, replaced hexagons and circles, Silvia and Barona (2009) found that only experts preferred curved stimuli to the angular ones. This latter result seems consistent with the data from Leder and Carbon (2005), who also found a higher preference for curvature among those participants characterized as experts in the arts—although, in that case, the difference was explained as an interaction between innovation and curvature, with expert participants being more conservative than non-experts.

All in all, these findings are a reminder that the influence of cultural factors in preference for curvature should not be overlooked. Whereas most published studies have had a tendency to tacitly assume that the observed results are indication of a universal phenomenon, the general lack of cross-cultural data begs for a cautious attitude towards such assumptions.

### An Evolved Preference

A nativist explanation of preference for curvature should be fairly easy to defend from a sensorimotor point of view, as derived from biological constitution. Still, it has been precisely among the leading studies concerned with appraisal that a possible evolutionary origin has been more openly and widely discussed. The reason for this is twofold: on one hand, as previously shown, most research on curvature has focused on the different emotional connotations conveyed by round and sharp lines and contours. The strong and pervasive link between sharpness and negative, threatening, or agitated feelings has led researchers to regard this association as an adaptation. On the other hand, most of the sensorimotor approaches have avoided discussing the evolutionary origins of how we perceive curvature.

Allen (1877) argued for a biological origin of the appreciation of curved lines, and many other aesthetic features, before the publication of any of the experiments noted in the preceding sections. He did so, nonetheless, only in general terms. Specific arguments for the adaptive value of the affective responses to sharp and curved contours were not put forth until the late 20th century, after the restoration of evolutionary thinking in sociology, anthropology, and psychology. In this line, Uher (1991) explicitly linked the widespread use of zigzag motifs among different cultures of the world to ancient environmental pressures. She found this pattern to be usually present in aggressive and defensive contexts, often accompanied by the symbolic representation of eye motifs, which have been found to produce an aversive reaction due to their threatening nature (Coss, 1972; Ellsworth et al., 1972). To explore this possibility, she presented 1100 participants from Central Europe with different sets of wavy and zigzag lines, some of these accompanied by eye motifs. She then asked participants to classify the stimuli according to 24 pairs of opposed adjectives. Consistent with her hypothesis and previous findings, Uher (1991) found that zigzag lines were reliably and significantly associated with antagonistic adjectives, whereas wavy lines were associated with affiliative ones. She found this evidence to be in favor of the influence of our biological heritage in the use of zigzag motifs; an influence that, nonetheless, was susceptible to cultural modulation. These considerations would later provide the backbone for Aiken’s (1998) argument that preference for curvature was actually motivated by a fear induced by sharp lines. This fear, she argued, had served an adaptive function in our distant past: to help us rapidly detect and avoid possible threats to our survival. Aiken (1998) believed this to be a canonical example of how primitive emotions, which originated initially as a fitness-increasing response to environmental pressures, have been repurposed, owing to their interaction with higher cognition, giving rise to the aesthetic experience of art.

But it was Bar and Neta (2007) who first tested the possible link between preference for curvature and the threatening nature of sharp lines. By taking in account subjective perceptions of preference and threat, as well as relative levels of amygdala activity, they found empirical support for the hypothesis that preference for curvature results from a primitive association between sharp transitions in contour and a sense of threat. Bar and Neta (2008), however, later interpreted their findings from a non-nativist point of view, as the result of developmental learning. Given the lack of cross-cultural data, and of a clearly defined evolutionary scenario in which environmental pressure would be sufficient to warrant such an adaptation, this is an equally plausible scenario.

Nevertheless, while these limitations invite us to be cautious, there are scattered data that support the idea of an evolutionary origin of preference for curvature, whether understood from a sensorimotor or an appraisal perspective. In particular, it has been found that not only children (Jadva et al., 2010), but even 1 week-old infants (Fantz and Miranda, 1975), have a tendency to look longer at curved stimuli than sharp ones. Furthermore, recent research has found that preference for curvature is also present in non-Western cultures, such as rural Ghana (Gómez-Puerto et al., 2013), and even among non-human primates (Munar et al., 2015).

Additionally, the idea that sharp angles are perceived as threatening due to evolutionary constraints has antecedents also in face research. Larson et al. (2007) showed that minimal geometric figures, resembling facial configurations of expressed anger and happiness, influence attentional processes and the attributed semantic meaning. Specifically, people associate angular V-shaped geometric figures—straight lines converging in an angle—to anger, and rounded shapes and figures to happiness (Aronoff et al., 1992; Aronoff, 2006). The authors posit that such configurations might be processed by Ekman’s (2003) “auto-appraisers”, a hypothesized set of feature detectors—innate appraisal mechanisms—that enable observers to quickly decode facial emotional expressions (Larson et al., 2007). Hence, the human preference for curvature might owe to a deep-rooted association between angular and curved geometric configurations and threatening and pleasant facial expressions, respectively. This, however, is not the only possible link between preference for curvature and facial features. Neotenic features—juvenile physical traits still present in adults—tend to result in salient curved configurations, such as a rounded head or large rounded eyes, and seem to have been favored by sexual selection. Neoteny and attraction toward neotenic traits, therefore, constitute plausible evolutionary foundations for preference for curvature (Bertamini et al., 2015).

In a different direction, LoBue (2014) has proposed that the widely studied Snake Detection Hypothesis—the idea that humans visually detect snakes faster than other stimuli—might be explained by the curvilinear body characteristic of those animals. By deconstructing snakes’ anatomy into its very basic curved features, she was able to compare the detection time of these features to that of its rectilinear equivalents, showing that the faster detection times reported when employing photographs of real snakes remain even when participants are presented with its most basic, curvilinear features.

All in all, there is a certain amount of evidence supporting a possible evolutionary origin of preference for curvature, and several unexplored hypotheses that could explain it. Humans might avoid sharp contours because they are related to threats in nature, or because we are hardwired to detect threatening facial expressions. But we might also be faster and better at processing curved stimuli, and prefer them because of this very same reason. Solid answers will only be provided by developing these proposals into testable hypotheses, and gathering data from different cultures and species.

## Further Research

There is enough evidence to consider preference for curvature as a well-established phenomenon and yet, after more than a century of research, we are still far from a good explanation. This is due to a lack of a strong, unifying framework and a common perspective, but also to the fact that several fundamental and pressing issues have still to be appropriately addressed.

First of all, there is a matter of conceptual and terminological clarity. As can be seen in Table 1, whereas there is an overall consensus in the use of terms in the domain of *curvature* (i.e., *curves*, *curved lines*, *curvy*), the nomenclature usually employed is far from univocal, specially when addressing the lack of curvature itself: is it angularity? Sharpness? Straightness? Broken, zigzag lines? Is it all the same? This question is far from trivial. We have mentioned how a widespread hypothesis postulates that preference for curvature is a result of the threatening appearance of sharp-angled contours, due to developmental or evolutionary reasons. An illustration of what researchers have in mind when making such claims can be found when Carbon (2010) presents the images of shark teeth, the outline of a shark, and a rose thorn as paradigmatical examples of sharp transitions in nature signaling threat. In these three instances, it could be argued that the stimuli, while pointy, are curved in contour, not sharp-angled. As a matter of fact, it might not be easy to find examples of strictly sharp-angled contours present in the organic environment of primates. Defining central concepts in a clear and univocal manner should be the first step to build compelling and testable explanations of preference for curvature.

**Table 1:** **Terminology used throughout relevant literature on curvature**.

Term	Opposed to	Reference
Curved, curves	Straight	Hogarth (1753), Stratton (1902), Lundholm (1921), Poffenberger and Barrows (1924), Hevner (1935), Fantz and Miranda (1975), Quinn et al. (1997) and Leder and Carbon (2005)
	Straight, waving, ellipses, circles	Martin (1906)
	Angular	Allen (1877), Guthrie and Wiener (1966), Kastl and Child (1968), Carbon (2010) and Bertamini et al. (2015)
	Pointed/sharp, zigzag	Aiken (1998)
	Sharp, sharp-angled	Allen (1877), Bar and Neta (2006), Jakesch and Carbon (2011), Leder et al. (2011) and Gómez-Puerto et al. (2013)
Round	Sharp	Larson et al. (2007), Hess et al. (2013) and Gómez-Puerto et al. (2013)
	Angular	Silvia and Barona (2009), Jadva et al. (2010) and Westerman et al. (2012)
Wavy, waving	Straight	Hogarth (1753)
	Straight, curved, ellipses, circles	Martin (1906)
	Zigzag	Uher (1991)
Curvilinear	Rectilinear	Vartanian et al. (2013) and LoBue (2014)
Serpentine	Straight	Hogarth (1753)

*While some authors make use of several terms as synonyms, we have only included those that appear in a consistent manner in a given work*.

But in order to achieve this, we might need to delve into the psychophysics of curvature. When is an angle perceived as sharp? What is deemed to be curved? Do different features of the stimuli (size, extension, complexity, dimensions) affect the perceived curvature? Actually, it is not even clear whether the phenomenon we are dealing with consists of a preference for curvature or an avoidance or rejection of sharpness. There is enough evidence supporting both hypotheses, and future research should attempt to clarify this apparent contradiction.

Furthermore, it has usually been assumed that preference for curvature is a fundamentally visual phenomenon. And yet, it is no surprise that the same preference has been found in the haptic domain, employing three-dimensional, physical objects (Jakesch and Carbon, 2011). As we have seen, in the early days of the study of the perception of curvature, isolated lines were the main objects of study. It was in the second half of the 20th century that abstract and geometrical forms, and the contour of real objects, began to be studied. Still, there is a need of further evidence proving that preference for curved contours is the same phenomenon as preference for isolated curved lines. Researchers considering this possibility should also address the fact that most research has usually involved bi-dimensional objects, and should consider why we expect the principal domain of such preference to be visual.

So far, most research has implicitly assumed the universality of the phenomenon, with only a few papers daring to suggest an evolutionary explanation in a couple of sentences. The most serious approach was Carbon’s (2010) attempt to prove the influence of culture in preference for curvature, which, in isolation, is incomplete. In this regard, research on curvature could benefit from following a theoretical development akin to that of the Snake Detection Hypothesis, a somewhat similar phenomenon that, as we have discussed, might even be related. A number of studies have shown over and over that humans of different ages are especially fast when detecting snake and snake-like stimuli. The use of standardized tests, together with strong and consistent findings, has led this theory to be widely accepted. But, as is still the case in research for curvature, the universality and evolutionary origin of snake detection was mostly taken for granted without further proof. It was not until recently that Isbell (2006) wrote a compelling case for its evolutionary implications, proposing up to 36 testable predictions derived from her hypothesis. These range from comparing speed differences in snake detection to the possibility of establishing datable paleotropical relationships through the study of retroviruses. Turning the hypothesis into a testable theory allowed for an enriching and strong debate that has benefited from the expertise of primatologists, evolutionary anthropologists, and other professionals with different interests other than the psychological aspects of the theory. We believe that further theoretical developments in preference for curvature should follow this example by expanding its current tentative explanations into full testable predictions, or by proposing new ones altogether. On a more basic level, delineating the evolutionary history of human preference for curvature requires gathering further relevant data from non-Western cultures, and other primates and mammals.

All these considerations form the foundations of a unified framework that aims to advance the understanding of preference for curvature. Future studies and theoretical work will undoubtedly shape it further. As a starting point, we recapitulate what we consider the most relevant conclusions that can be reached from published research:

- People tend to prefer curved stimuli to sharp-angled ones. This phenomenon has been studied mostly in Western populations, but it has also been found in newborn babies, non-Western cultures, and other primates. Thus, there is some evidence supporting the universality or innateness of this preference. Nevertheless, other evidence suggests that it is culturally influenced. The two options are not mutually exclusive, but they should be acknowledged and considered when designing and discussing further research.
- This phenomenon seems to encompass isolated lines as well as shapes and contours. Still, it is not proven whether the similarities imply the same underlying mechanism. Moreover, it is also unclear whether this is a unimodal or multimodal phenomenon.
- The term *curvature* is widely used throughout most published research. Deeming it wise to reach some terminological consensus in order to strengthen the field and avoid misunderstandings, we propose the use of the dichotomy *curvature/sharpness* to describe the object of study, and *curved/sharp-angled* to characterize the stimuli causing the effect. There is a case to be made for the use of *angular* as opposed to *curved*, but its polysemy might be misleading. The feature of interest is not the number of angles, but the degree of their curvature.
- These conceptual quandaries clearly show that the psychophysical nature of these features is yet to be explored. For now, any stimulus whose angles are evidently smooth, forming a continuous line that is perceived as such in plain view should be understood as curved.
- There is not enough evidence to ascertain whether we are indeed dealing with a preference for curvature or an avoidance or rejection of sharpness. So far, we consider it more parsimonious to speak of preference, but this issue requires further study. When proposing an evolutionary origin, it is not enough to suggest a plausible explanation. A detailed scenario with testable predictions is required for the hypothesis to be useful.

The study of preference for curvature is a promising endeavor. Not only because of its aesthetic, psychological, evolutionary, and epistemological implications, but also because of its practical consequences. One of humans’ defining characteristics is the extent to which we create and shape our environment, and the freedom of choice we have in doing so. We surround ourselves with stimuli differing in curvature. And there is evidence showing how much this feature affects our perception, preference, and choice of—for instance—cars (Leder and Carbon, 2005), products’ graphics and container designs (Westerman et al., 2012), and architectural interiors (Vartanian et al., 2013). Not only do we prefer curved-contoured objects, but we also find them more innovative, less aggressive, and we are more willing to purchase them. Moreover, curved and sharp elements in our environment might even influence decisions about cooperating and competing with others (Hess et al., 2013).

In sum, the contours of objects around us and with which we interact are not mere inconsequential design niceties. They have a tangible impact on our preferences and choices in consumer and social contexts. A better understanding of the mechanisms underlying preference for contour, and their evolutionary and cultural foundations, will therefore contribute to explaining human behavior in such contexts.

## Conflict of Interest Statement

The authors declare that the research was conducted in the absence of any commercial or financial relationships that could be construed as a potential conflict of interest.
